# HCN Channels Are Not Required for Mechanotransduction in Sensory Hair Cells of the Mouse Inner Ear

**DOI:** 10.1371/journal.pone.0008627

**Published:** 2010-01-07

**Authors:** Geoffrey C. Horwitz, Andrea Lelli, Gwenaëlle S. G. Géléoc, Jeffrey R. Holt

**Affiliations:** Department of Neuroscience and Department of Otolaryngology, University of Virginia School of Medicine, Charlottesville, Virginia, United States of America; The Research Center of Neurobiology-Neurophysiology of Marseille, France

## Abstract

The molecular composition of the hair cell transduction channel has not been identified. Here we explore the novel hypothesis that hair cell transduction channels include HCN subunits. The HCN family of ion channels includes four members, HCN1-4. They were orginally identified as the molecular correlates of the hyperpolarization-activated, cyclic nucleotide gated ion channels that carry currents known as I_f_, I_Q_ or I_h_. However, based on recent evidence it has been suggested that HCN subunits may also be components of the elusive hair cell transduction channel. To investigate this hypothesis we examined expression of mRNA that encodes HCN1-4 in sensory epithelia of the mouse inner ear, immunolocalization of HCN subunits 1, 2 and 4, uptake of the transduction channel permeable dye, FM1-43 and electrophysiological measurement of mechanotransduction current. Dye uptake and transduction current were assayed in cochlear and vestibular hair cells of wildtype mice exposed to HCN channel blockers or a dominant-negative form of HCN2 that contained a pore mutation and in mutant mice that lacked HCN1, HCN2 or both. We found robust expression of HCNs 1, 2 and 4 but little evidence that localized HCN subunits in hair bundles, the site of mechanotransduction. Although high concentrations of the HCN antagonist, ZD7288, blocked 50–70% of the transduction current, we found no reduction of transduction current in either cochlear or vestibular hair cells of HCN1- or HCN2- deficient mice relative to wild-type mice. Furthermore, mice that lacked both HCN1 and HCN2 also had normal transduction currents. Lastly, we found that mice exposed to the dominant-negative mutant form of HCN2 had normal transduction currents as well. Taken together, the evidence suggests that HCN subunits are not required for mechanotransduction in hair cells of the mouse inner ear.

## Introduction

Although biophysical evidence revealed the existence of mechanosensitive ion channels in hair cells of the vertebrate inner ear over 30 years ago [1], the molecular composition of the channels remains a mystery. Furthermore, the auditory and vestibular systems are among the few sensory systems for which the primary transduction channels have not been identified. Conventional approaches designed to reveal the identity of the hair cell transduction channel based on examining candidates with similar channel properties have proven fruitless thus far, suggesting that a broader perspective may be required. In that spirit we opted to explore the recent novel hypothesis which suggested that the hair cell transduction channel may include members of the HCN channel family [2]. Ramakrishnan et al. [2] found several lines of evidence that supported their hypothesis including expression of HCN1 mRNA in hair cells of trout vestibular organs and mammalian cochlea, immunolocalization of HCN1 protein in sensory hair bundles and a calcium-dependent interaction between HCN1 and the putative tip link molecule protocadherin-15. Since protocadherin-15 has been localized at the lower end of tip-links [3] and a recent calcium imaging study placed transduction channels at the lower end of tip-links [4], the interaction between protocadherin-15 and HCN1 would place the HCN1 subunit in the correct location to mediate hair cell transduction. We also note that Lin et al. [5] demonstrated that HCN channels are sensitive to mechanical stretch and that hair cell transduction has been reported to be sensitive to cyclic nucleotides [6], one of several features that characterize HCN channels. These observations raise the profile of HCN channels as potential candidates for a role in hair cell mechanotransduction.

Previous work has focused on HCN subunits as the molecular correlates of hyperpolarization-activated, cyclic nucleotide modulated currents referred to as I_f_, I_Q_ or I_h_. There are four members of the HCN channel family, HCN1-4, each of which has six transmembrane domains and a single pore-forming domain. They have been shown to form functional ion channels either as homo- or hetero- tetramers. HCN channels are permeable to sodium, potassium and calcium [7] with reversal potentials that range from −40 to −10 mV and very small single channel conductances, ≤1.5pS [8]. Hair cell transduction channels are also cation-selective with permeability to potassium, sodium and calcium and a reversal potential close to 0 mV but have little voltage dependence [9] and a large single channel conductance, >100 pS [10]. Although there are some striking differences between the biophysical properties of HCN currents and those of hair cell transduction currents, it is possible that the biophysical properties of HCNs differ significantly depending on the binding partners with which they interact and the local environment in which they are expressed. For example, HCNs exposed to a high external potassium solution, such as endolymph which bathes hair bundles, have a more positive reversal potential [11] and may have a higher single-channel conductance as well.

Despite the differences between hair cell transduction currents and HCN currents and considering the lack of progress toward identification of the hair cell transduction channel, we felt the Ramakrishnan et al. [2] hypothesis warranted further investigation. As such, we opted to explore the possibility that HCN subunits contribute to mechanotransduction in both auditory and vestibular hair cells of the mouse inner ear. We examined mRNA expression, protein localization, uptake of fluorescent dyes and mechanotransduction currents evoked by hair bundle deflections. Experiments were performed on wild type mice and mice deficient in HCN1, HCN2 or both and wild-type mice transfected with mutant HCN2. We found little evidence to support the Ramakrishnan et al. [2] hypothesis and conclude that HCN subunits are not required for hair cell transduction.

## Results

### Expression of HCN mRNA and Protein

**Figure 1:**
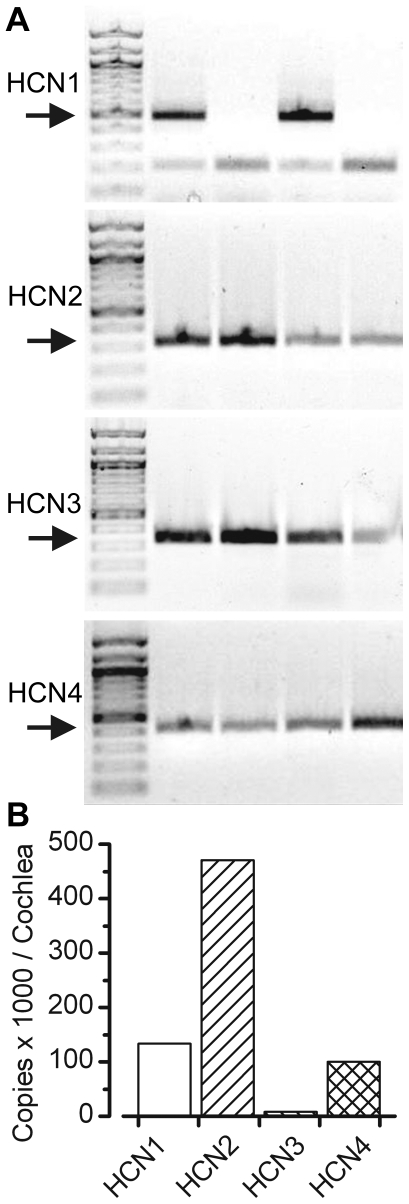
Expression of HCN mRNA. (A) Conventional RT-PCR was used to examine the expression of HCN1-4 in vestibular epithelia of wild-type mice. Arrows indicate the expected size of the PCR product: HCN1: 491 bp; HCN2: 337 bp; HCN3: 339 bp and HCN4: 419 bp. Lane 1 in each gel contained markers. Each subsequent lane contained the PCR product obtained using template cDNA harvested from the following sources. Lane 2: wild-type mouse brain; Lane 3: HCN1-deficient mouse brain; Lane 4: wild-type utricle; Lane 5: HCN1-deficient mouse utricle. (B) Quantitative RT-PCR was used to estimate total number of copies of messenger RNA for each of the four HCN subunits using wild-type mouse cochlea as template. Total copies in thousands of HCN mRNA transcripts per cochlea. Results were from a pool of 4 cochlea obtained from mice at P4.

We began with a molecular screen for members of the HCN family. Conventional RT-PCR data revealed that all four HCN subunits were expressed in the vestibular organs harvested from the mouse inner ear at P8 (Figure 1A). To examine relative expression levels of HCN subunits we used quantitative RT-PCR with specific, validated primer sets and mRNA extracted from the sensory epithelia of four mouse cochlea at P4 (Fig. 1B). Consistent with a previous report [12], we found HCN 1 and 2 were the most abundant and HCN3 was the least abundant. The expression ratios for HCN1-4 were 18∶62∶1∶13. We also examined relative expression in the cochleas of mice that were deficient for both HCN1 and HCN2. In this case, we were unable to detect a signal for HCN1 or HCN2 and found that the relative expression of HCN3 and HCN4 was similar to wild-type: 0∶0∶1∶23.

**Figure 2:**
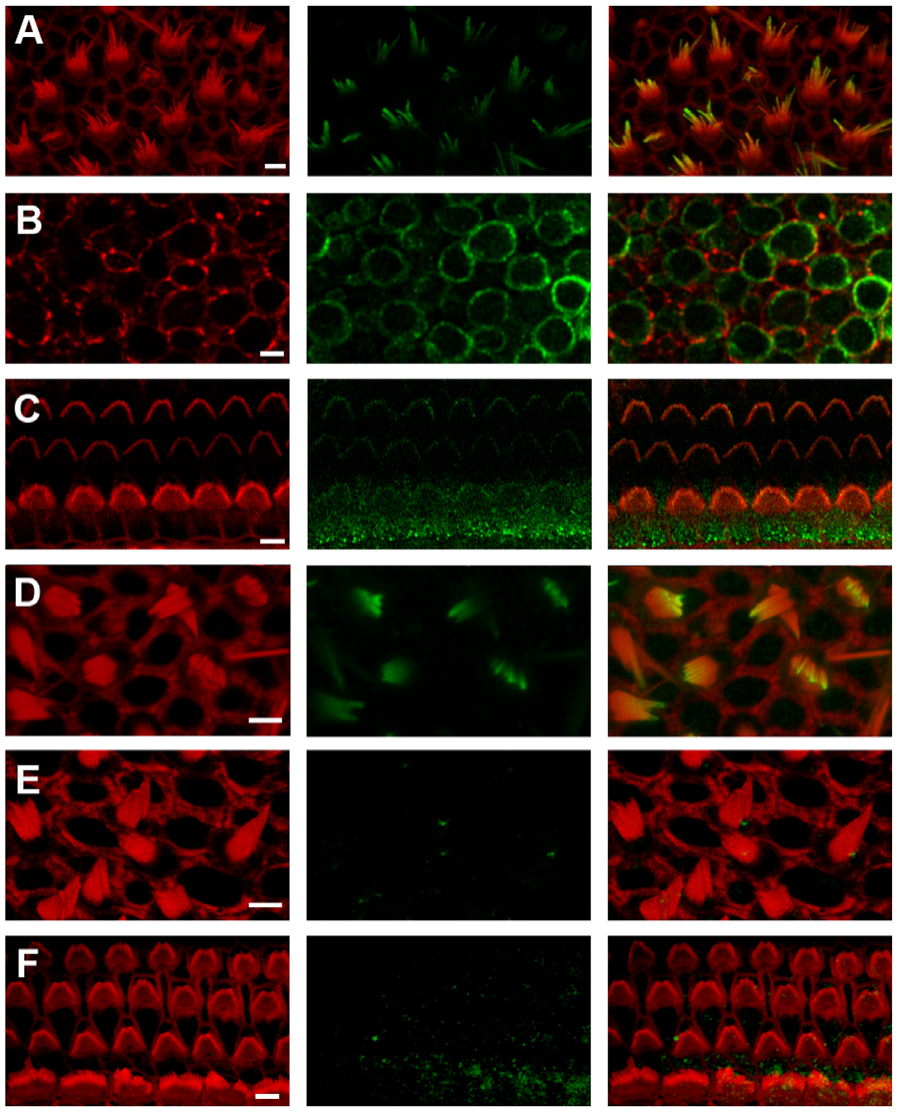
Immunolocalization of HCN subunits in the inner ear. All panels show confocal images of mouse inner ear epithelia with phalloidin staining shown on the left, HCN1 in the center and the merged image on the right. Phalloidin is shown in red and HCN1 in green. All scale bars indicate 5 µm. (A) Stereociliary bundles of wild-type mouse utricle at P8 stained with an antibody directed against the N-terminus of HCN1. (B) Basolateral hair cell membranes of wild-type mouse utricle stained with the N-terminal HCN1 antibody. (C) Stereociliary bundles of wild-type mouse cochlea harvested from the apex at P8 stained with same N-terminal HCN1 antibody. (D) Confocal image of the stereociliary bundles from a P8 utricle of a HCN1^−/−^ mouse stained the same HCN1 antibody shown in panels A–C. (E) Wild-type utricle focused at the hair bundle level stained with a different antibody that recognizes an epitope in the C-terminus. (F) Wild-type cochlear hair bundles stained with the antibody that recognizes the epitope in the C-terminus.

Since the tissue samples from which the mRNA was extracted included hair cells, supporting cells and nerve terminals we were interested to localize the cell type in which each HCN subunit was expressed as well as the subcellular localization within hair cells with particular attention focused on the sensory hair bundles. In mouse utricles we found immunoreactivity in hair bundles (Fig. 2A) and the basolateral membranes of the hair cells (Fig. 2B) using an antibody directed against the N-terminus. In the cochlea HCN1 immunoreactivity was restricted to the hair bundles of both inner and outer hair cells (Fig. 2C) but was absent from the basolateral membranes. This pattern of HCN1 immunoreactivity in both vestibular and auditory hair bundles was consistent with that reported by Ramakrishnan et al. [2] using the same antibody directed against the N-terminus. However, upon further investigation we found that the hair bundle signal was also present in auditory and vestibular hair cells (Fig. 2D) of HCN1-deficient mice. The cell body staining was absent (not shown), which lead us to suspect that the hair bundle signal may have resulted from non-specific binding of the primary antibody.

When we used a different primary antibody, one directed against an epitope near the C-terminus of the HCN1 protein, the localization was restricted to the basolateral membranes of wild-type vestibular hair cells (not shown) with no immunoreactivity in vestibular (Fig. 2E) or cochlear hair bundles (Fig. 2F). The vestibular basolateral membrane staining was absent in the HCN1-deficient mice.

We detected no immunoreactivity for HCN2 or HCN4 in either auditory or vestibular hair cells, though both were detected in the afferent neurons that innervate inner ear organs (not shown). The specificity of the HCN2 antibody was confirmed in HCN2-deficient mice as these mice lacked the afferent localization pattern that was present in the wild-type neurons that innervate the inner ear.

### Mechanotransduction Currents

Although we did not observe HCN subunit localization in hair bundles, it remained possible that very low levels of expression, below our detection threshold, were present and sufficient for normal function. Indeed, transduction models suggest that as little as one ion channel per stereocilium, below the detection limit of conventional immuno-fluoresence techniques, may be sufficient to confer functional mechanotransduction. As such, given the expression of HCN mRNA in auditory and vestibular epithelia and the putative interaction between HCN1 and protocadherin-15 [2] we wondered whether disruption of HCN function would cause dysfunction in hair cell transduction.

**Figure 3:**
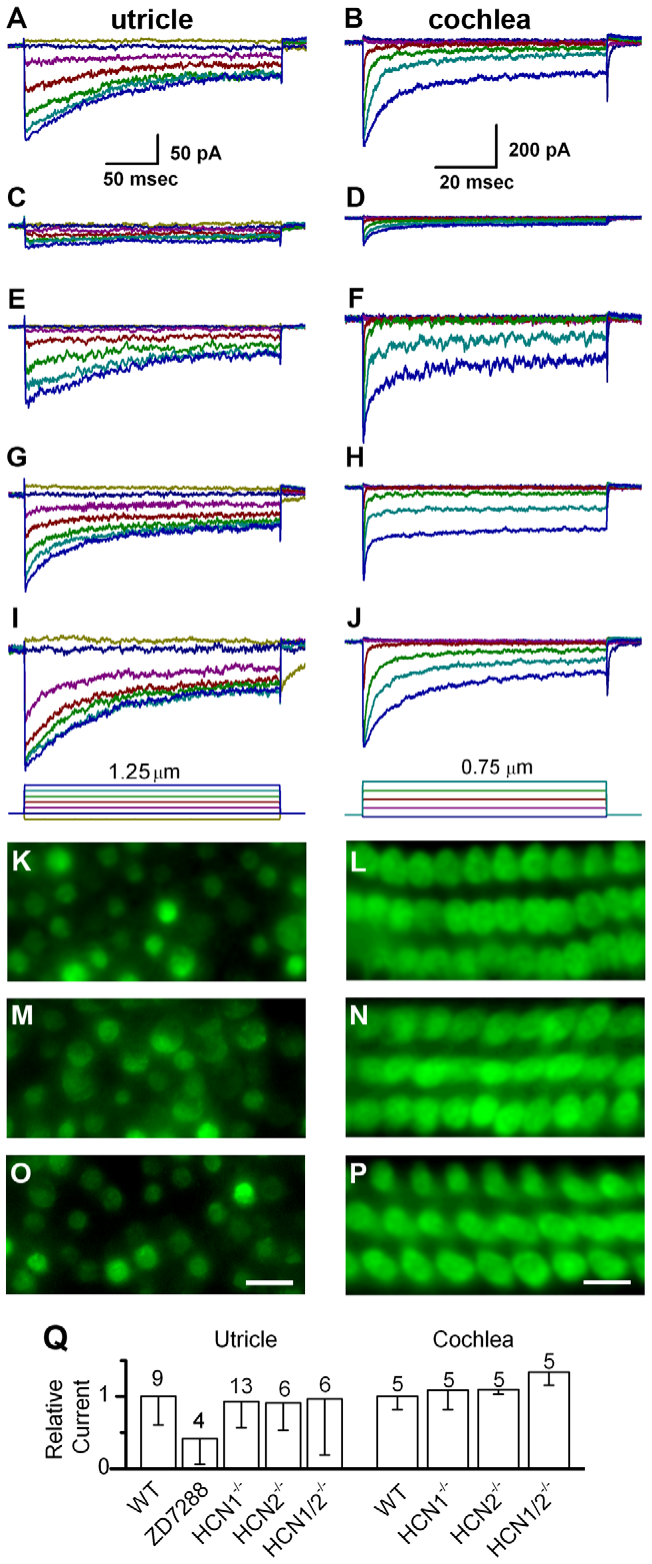
Mechanotransduction in wild-type and HCN-deficient hair cells. Representative mechanotransduction currents evoked by hair bundle deflections. The left column of data were recorded from mouse utricle type II hair cells at P3 - P6. The data in the right column were recorded from mouse cochlear outer hair cells at P6 - P7. The scale bars at the top apply to all data in the column. The deflection protocol is shown at the bottom of trace panels I and J. Data were recorded under the following conditions: (A & B) wild-type control; (C & D) in the presence of 500 µM ZD7288; (E & F) HCN1^−/−^; (G & H) HCN2^−/−^; (I& J) HCN1^−/−^ and HCN2^−/−^. Panels K-P show fluorescent images of hair cells following application of the transduction channel permeable dye, FM1-43, in utricle (P5 –P7) and organ of Corti (P6 – P9) sensory epithelia under the following conditions: (K & L) HCN1^−/−^; (M & N) HCN2^−/−^; (O& P) HCN1^−/−^ and HCN2^−/−^. Uptake of FM1-43 appeared normal in all tissues examined. The scale bar in panel (O) equals 10 µm and also applies to panels K and M. The scale bar in panel (P) equals 5 µm and also applies to panels L and N. (Q) Summary of transduction currents recorded from vestibular and auditory hair cells. Maximum current amplitudes under each condition were averaged and were normalized relative to wild-type controls. Error bars show standard deviation; the number of samples is indicated above each bar.

To assay for changes in hair cell mechanosensation we began by recording transduction currents evoked by hair bundle deflections while manipulating HCN function. Several antagonists have been identified that inhibit HCN function. To test whether hair cell transduction was affected by HCN antagonists we applied 500 µM ZD7288 to acutely excised mouse utricle and cochlea epithelia and deflected hair bundles. Transduction currents were recorded using the whole-cell, tight-seal technique in voltage-clamp mode. Relative to wild-type control currents (Fig. 3A & 3B), we found that hair cells exposed to ZD7288 had 58% less transduction current in vestibular hair cells (Fig. 3C) and 70% less current in cochlear outer hair cells (Fig. 3D). These data were consistent with the notion that the hair cell transduction channel includes HCN subunits. However, ZD7288 may have non-specific effects, particularly at the high concentrations used here. At lower concentrations, 100 µM ZD7288, the effect was less pronounced. Since hair cell transduction is susceptible to blockade by a large number of non-selective compounds [13] we sought a more selective assay. We recorded transduction currents from mice deficient in either HCN1 (Fig. 3E & 3F) or HCN2 (Fig. 3G & 3H). Both auditory and vestibular hair cells had transduction currents that resembled wild-type currents in every respect. We recorded normal transduction from 29/29 hair cells deficient in HCN1 or HCN2 (Fig 3Q). Since mechanotransduction currents must be assayed one cell at a time we also applied the transduction channel permeable dye FM1-43 which allowed us to visually assay transduction channel activity in thousands of hair cells from all regions of the mouse utricle and cochlea. In previous work, we demonstrated that FM1-43 uptake can be abolished in mouse hair cells by bath application of the transduction channel blocker gentamicin [14]. Dye uptake in hair cells of both HCN1- and HCN2- deficient mice was indistinguishable from wild-type hair cells throughout auditory and vestibular epithelia (Fig. 3K–N). Taken together the normal transduction currents and normal dye uptake ruled out an essential role for either HCN1 or HCN2 in hair cell mechanotransduction. To address the possibility that either HCN1 or HCN2 could compensate for the loss of the other we generated mice deficient in both genes. We found that hair cells from mice that lacked both HCN1 and HCN2 also had normal transduction currents (Fig. 3I & 3J) and normal FM1-43 uptake (Fig. 3O & 3P), which ruled out any possibility that either subunit is necessary for mechanotransduction. The transduction data from all 58 hair cells are summarized in Figure 3Q.

**Figure 4:**
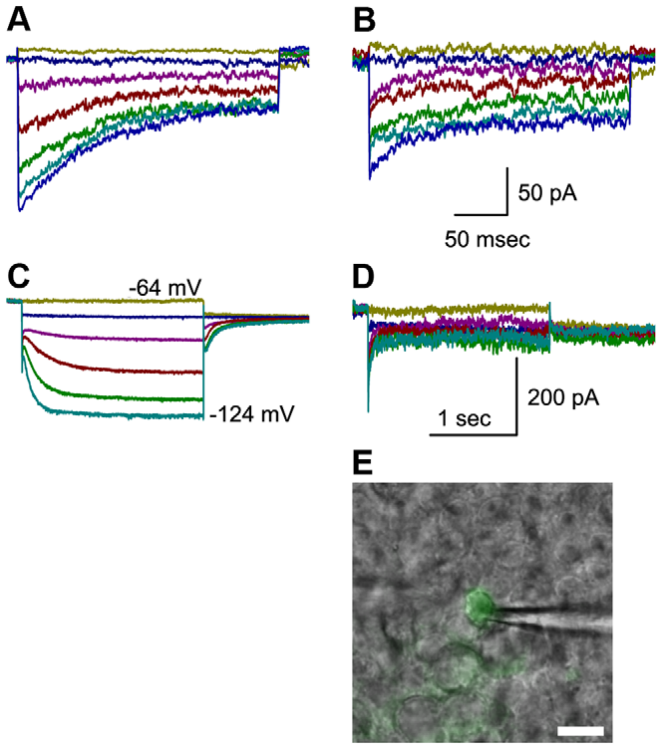
Whole-cell currents recorded from control and transfected cells. (A & B) Representative mechanotransduction currents recorded from mouse utricle type II hair cells at P3 - P6. Bundle deflections were evoked using the protocol shown at the bottom of figure 3I. Panel A shows data from a non-transfected control cell and panel B shows data from a GFP+ cell transfected with the HCN2-AYA construct. The scale bars in B apply to panels A and B. (C & D) Representative currents recorded in response to families of voltage steps that ranged between −124 mV and −64 mV in 10 mV increments. Capacitive transients and leak currents were subtracted for clarity. The scale bars in panel D apply to both panels C and D. Panel C shows I_h_ recorded from a non-transfected utricle type II hair cell from the same epithelium as that shown in panel A. Panel D shows a family of currents recorded from the same GFP+ shown in panel B. A family of voltage steps was used that was identical to those used to evoke the data shown in panel C. In this case, expression of the HCN2-AYA construct inhibited I_h_. (E) A fluorescence image that revealed GFP expression was superimposed on a DIC image of the same field of cells. The recording pipette is visible to the right of the cell. Scale bar equals 5 µm.

We still wondered whether there might be some involvement of HCN 3 or 4. However, since HCN3 expression was extremely low we ruled it out for further consideration. Since mice deficient in HCN4 die at embryonic stages [15] before the formation of hair cells, ∼E12, it was not possible to examine transduction in HCN4-deficient hair cells. As an alternative approach, we decided to test for involvement of HCN4 by transfecting mouse utricle cultures with an adenoviral vector that carried a mutant form of HCN2 [16]. Previously, an AYA substitution was made that replaced the GYG sequence in the pore-forming region which resulted in dominant-negative suppression of HCN4 [16]. Since HCN2 subunits are able to coassemble with HCN1, 2 and 4 subunits [17], we reasoned that exogenous dominant-negative HCN2 should be able to coassemble and suppress the function of any of its coassembly partners expressed endogenously. We recorded normal transduction currents in 3/3 utricle hair cells transfected with mutant HCN2 (Fig. 4A&B). As a positive control for the adenoviral transfection, expression of HCN2 AYA, and coassembly with endogenous HCN subunits we recorded voltage-dependent currents in type II vestibular hair cells and found a significant reduction in voltage-dependent I_h_ (Fig. 4D)

## Discussion

The search for the elusive hair cell transduction channel continues. Based on the data presented here we now include HCN channels on the growing list of ion channel genes which can be ruled out for further consideration as transduction channel candidates. The lack of a hair cell mechanotransduction deficit in HCN1 knockouts, HCN2 knockouts, double knockouts for HCN1 and HCN2 and in hair cells transfected with the dominant-negative construct HCN2 AYA, along with the low expression levels of HCN3 leads us to conclude that HCN subunits are not necessary for hair cell transduction. In consideration of this result and other recent progress, we suggest that the molecular identity of the hair cell transduction may ultimately be revealed by process of elimination.

While the hypothesis that hair cell transduction may require HCN subunits, particularly HCN1, was based on some sound arguments [2] we found that some of the evidence was refutable. In particular, we found no convincing data to support immunolocalization of HCN1 to auditory and vestibular hair bundles. We used an antibody directed against the N-terminus of HCN1, identical to the one used by Ramakrishnan et al. [2] to localize HCN1 in rat cochlea, but conclude that the hair bundle staining was the result of a non-specific interaction, since the same localization pattern was present in hair bundles of wild-type and HCN1 knockout mice. A second more selective antibody, directed against the C-terminus, revealed HCN1 localization within the basolateral membranes of vestibular hair cells but not in hair bundles. HCN1 staining was not identified in basolateral membranes or hair bundles of cochlear hair cells. Both antibodies revealed that the basolateral localization of HCN1 was absent in vestibular hair cells of HCN1 knockout mice, which suggested the basolateral staining was an accurate representation of HCN1 localization in hair cells. As such, we conclude that hair bundle localization pattern reported by Ramakrishnan et al. [2] may have been in error due non-specific binding of the N-terminal antibody to non-HCN1 epitopes present in hair cell stereocilia. Our PCR data was consistent with that of Ramakrishnan et al. [2] and confirmed expression of HCN1, 2 and 4 in both auditory and vestibular epithelia and very low expression of HCN3. Furthermore, we saw no significant difference in HCN3 or HCN4 expression between wild-type mice and mice deficient for HCN1 and HCN2 suggesting that upregulation of the subunits that remain does not occur in the mouse cochlea. We did not investigate the reported interaction between HCN1 and protocadherin-15.

Although our qPCR screen suggested that HCN1, HCN2 and HCN4 are expressed in the cochlea, we note that the tissue samples from which the RNA was harvested included multiple cells types and perhaps spiral ganglion neurons which are known to express HCN subunits [18]. Despite the strong qPCR signal, the lack of HCN protein localization in cochlear hair cells, the lack of transduction deficits in cochlear hair cells, the lack of an auditory phenotype in HCN knockout mice [19] and the lack of I_h_ in wild-type cochlear hair cells leads us to conclude that HCN channels do not contribute to the normal function of postnatal auditory hair cells.

The role of HCN channel function in vestibular hair cells remains to be determined. Although there was no transduction phenotype in vestibular hair cells of HCN1-, HCN2-, double mutants, or cells transfected with HCN2 AYA, we found strong evidence supporting expression of HCN protein in vestibular hair cells. Furthermore, previous reports have documented physiological expression of the hyperpolarization-activated current, I_h_, in mouse utricle hair cells [20] and here we show that I_h_ can be suppressed by the HCN2 AYA construct. Which HCN subunits carry I_h_ in mouse vestibular hair cells and their contribution to sensory signaling and vestibular function will require further investigation.

## Materials and Methods

### Tissue Preparation

Protocols approved by the Animal Care Committee of the University of Virginia (Protocol #3123) were used to harvest the organ of Corti from Swiss Webster mice (Hilltop Lab Animals Inc., Scottdale, PA and Taconic Farms, Germantown, NY) or B6129SF2/J mice (The Jackson Laboratory, Bar Harbor, ME) as controls at postnatal day (P) 0 to P8. Mouse pups were killed by rapid decapitation. The temporal bones were excised and bathed in MEM (Invitrogen, Carlsbad, CA) supplemented with 10 mM HEPES (pH 7.4). Utricle and cochlea sensory epithelia were gently dissected and mounted as previously described [21], [22]. A pair of thin glass fibers glued to the coverslip was placed on the edge of the tissue to stabilize it in a flat position. The tissue was used acutely for electrophysiological studies, fixed and prepared for immunocytochemistry as described below or placed in culture. Cultures were bathed in MEM with glutamax, supplemented with 10 mM HEPES, 25 mg/500 mL Ampicllin and 5% FBS and maintained in 5% CO_2_ at 37°C for up to 6 days as indicated. For some experiments adenoviral vectors were applied directly to the culture media at titers that ranged from 10^6^ to 10^8^ viral particles/mL. Adenoviral vectors that carried the HCN2 AYA coding sequence were obtained from Er et al. [16].

### RT-PCR

Total RNA was rapidly isolated with an RNA-aqueous kit (Ambion, Austin, TX) from sensory epithelia of ten utricles harvested from five P8 mouse pups. The isolated RNA was treated with a Turbo DNA-free kit (Ambion, Austin, TX) to eliminate genomic DNA. Isolated RNA was reverse transcribed into cDNA with random hexamer primers using the iScript kit (Bio-Rad, Hercules, CA). cDNA was used as template in RT-PCR reactions using the MasterTaq kit according to manufacturer specifications (Eppendorf, Hamburg, Germany). The following primer sets were designed to selectively amplify HCN1-4 mRNA and span introns to minimize amplification of genomic DNA: HCN1- TCAAGGAGGCAGTATCAAGAGAAG and ACCGAAAGGGAGTAAAGACGAC; HCN2 – ACTGCCCGCTGACTTCC and ATCTCCTTGTTGCCCTTGGT; HCN3– CACCGCCCTCATCCAGTC and CCCTCACGCACCACCAG; and HCN4 – TTTCATCTCCTCCATCCCTGTC and CCTGCCGTCCATACCCAAT. Amplification products were run on 1% agarose gels with Ethidium Bromide and were imaged using UV light and a ChemiImager (Alpha Innotech, San Leandro, CA). Products of the expected size were excised and purified using the quick extract gel kit (Qiagen, Valencia, CA). Purified PCR products were sequenced to verify the specificity of each reaction.

### Quantitative PCR

Total RNA was isolated as above from 1–2 P4 CD-1 mice. RNA concentration was measured on a spectrophotometer (Nandodrop, ND1000, Thermo Fisher Scientific, Pittsburgh, PA). To insure the quality of isolated RNA, representative samples were analyzed with a Bioanalyzer (Agilent Technologies, Santa Clara, CA) and found to have an RNA integrity number of >8.0. 400 ng of RNA was reverse transcribed into cDNA as described above. Quantitative PCR primers were designed using the PrimerQuest Software from Integrated DNA Technologies (San Diego, CA). Primers were designed with melting temperatures near 61°C. The primer sequences were: HCN1 – ACATGCTGTGCATTGGTTATGGCG and AACAAACATTGCGTAGCAGGTGGC; HCN2 – ACTTCCGCACCGGCATTGTTATTG and TCGATTCCCTTCTCCACTATG AGG; HCN3 – CCTCATCCGCTACATACACCAGT and GACACAGCAGCAACATC ATTCC; HCN4 – GCATGATGCTTCTGCTGTGTCACT and TTCACCATGCCATTG ATGGACACC. Each sequence was designed to amplify a region nested within an amplicon produced from RT-PCR, and was screened using a BLAST inquiry to verify specificity. The RT-PCR primers used to generate the amplicons were HCN1 – ACAAGACAGCCAGAGCACTTCGTA and CAGCAACACCGTGCTGGATGAAAT; HCN 2 – TCAACAAGTTCTCCCTGCGGATGT and TCAGCCAGATGTCTGTCATGCTCT; HCN3 – TTTCTTCCTGCTGGATCTGGTGCT and TAACACGTGGCGCCCACAATCATA; HCN4 - ATCGTGGTGGAGGACAACACAGAA and GAAACGCAACTTGGTCAGCATGGA. Single-color real time PCR reactions were designed and carried out in triplicate according to manufacturer specifications using iQ SYBR Green Supermix (BioRad, Hercules, CA) and run on an iCycler (BioRad Hercules, CA). Amplicons were generated for 40 cycles, and specificity was confirmed using melt curve analysis.

Standard curves were generated for each primer set in order to calculate total copy number and test for primer efficiency. In short, purified products from RT-PCR reactions described above were ligated into a pGEM-T Easy Vector (Promega, Madison, WI), cloned into competent *E. Coli* (New England BioLabs, Ipswich, MA) and concentrated using miniprep (Invitrogen, Carlsbad, CA). Concentration was obtained using a spectrophotometer (Nanodrop), and total number of plasmids/µL was calculated. standard curves for each primer set were generated by varying starting quantity between 300 and 300,000 total copies. Only primer sets which amplified efficiently (100%±5%) were used. Standard curves were as follows: HCN1 – Y = −3.178x+39.245; HCN2 - Y = −3.242x+38.617; HCN3 - Y = −3.229x+36.128, HCN4 - Y = −3.237x+38.446 where Y is the cycle threshold and X is the log of the starting quantity.

### Immunocytochemistry

Utricles were excised from B6129SF2/J (The Jackson Laboratory, Bar Harbor, ME), B6;129-HCN1^tm2Kndl^/J (The Jackson Laboratory, Bar Harbor, E), and HCN2^−/−^ mouse pups between P4 and P9. The epithelia were placed on glass coverslips under two glass fibers and were bathed in MEM with Glutamax (Invitrogen, Carlsbad, CA) buffered with 10 mM HEPES (Sigma, St. Louis, MO). The tissue was then placed in fixative containing 4% paraformaldehyde in 0.1 M phosphate buffered saline (PBS) for 15–20 minutes. Following fixation, the tissue was rinsed in PBS, incubated in 0.1% TritonX100 for 15 minutes, and blocked for one hour using a 3% bovine serum albumin and normal donkey serum. Incubation with primary antibodies was done overnight at 4°C. For HCN1, two primary antibodies were used: a rabbit polyclonal antibody directed against the N-terminus (Alomone, Israel, APC-056, 1∶200 dilution) and a goat polyclonal antibody directed against the C-terminus (Santa Cruz, Santa Cruz, CA, sc-19706, 1∶50 dilution). For HCN2, the primary antibody was a rabbit polyclonal directed against the N-terminus (Alomone, Israel, APC-030, 1∶100 dilution). The tissue was then incubated with Alexa Fluor secondary antibodies and Phalloidin (Invitrogen, Carlsbad, CA). Samples were imaged using a 63x oil-immersion objective on a Zeiss LSM510 confocal microscope (Oberkochen, Germany). Images were acquired using identical gain, contrast, pinhole, and laser intensity settings. Three-dimensional projection images were created following Z-series acquisition using the Zeiss LSM Image Browser.

For some experiments, the styryl dye FM1-43 (Invitrogen, Carlsbad, CA) was applied to the mounted tissue at 5 µM for 10 sec. The dye that partitioned into the outer leaflet of the cell membrane was washed out during three full bath replacements. Hair cells were imaged 5 minutes after exposure to FM1-43 using Axioskop FS plus microscope (Zeiss, Oberkochen, Germany) and standard fluorescence microscopy with an FM1-43 filter set. Identical gain and contrast settings were used for all image acquisition.

### Electrophysiological Recording

Recordings were performed in standard artificial perilymph solution containing (in mM): 137 NaCl, 0.7 NaH_2_PO_4_, 5.8 KCl, 1.3 CaCl_2_, 0.9 MgCl_2_, 5.6 D-glucose, and 10 HEPES-NaOH, adjusted to pH 7.4. Vitamins (1∶50) and amino acids and Glutamax (1∶100) were added from concentrates (Invitrogen, Carlsbad, CA). Hair cells were viewed from the apical surface using an upright Axioskop FS microscope (Zeiss, Oberkochen, Germany) equipped with a 63X water immersion objective with differential interference contrast optics. Recording pipettes (2–5 MΩ) were pulled from R-6 capillary glass (Garner Glass, Claremont, CA) and filled with intracellular solution containing (in mM): 135 KCl, 5 EGTA-KOH, 10 HEPES, 2.5 K_2_ATP, 2.5 MgCl_2_, 0.1 CaCl_2_, pH 7.4. Currents were recorded from vestibular type II cells or cochlear outer hair cells under whole-cell voltage-clamp at room temperature using either an Axopatch 200B or a Multiclamp 700A (Molecular Devices, Palo Alto, CA), filtered at 10 kHz with a low pass Bessel filter, digitized at ≥20 kHz with a 12-bit acquisition board (Digidata 1322) and pClamp 8.2 (Molecular Devices, Palo Alto, CA) and stored on disk for offline analysis using Origin 6.1 (OriginLab, Northampton, MA). Results are presented as means±S.E.M.

### Mechanical Stimulation

Vestibular type II hair cells were stimulated by drawing the kinocilium into a pipette filled with extracellular solution and held in place with suction [21]. Movement of the stimulus pipette was driven by a piezoelectric device that had a 10–90% rise time of 0.6 ms. Hair bundle deflections were monitored using a C2400 CCD camera (Hamamatsu, Japan). Voltage steps were used to calibrate the motion of the stimulus probe ±2 µm relative to its rest position. Video images of the probe were recorded to confirm absence of off-axis motion and calibrate the probe motion (spatial resolution of ∼4 nm). The position of the pipette and bundle were continuously monitored before, during and after each recording using video microscopy to ensure that the stimulus pipette and the hair bundle moved in unison.

Auditory outer hair cells were stimulated as previously described [22], [23]. Briefly, stiff glass probes were fire polished and shaped to fit into the concave aspect of the outer hair cell stereocilia. Deflections were evoked using a PICMA chip actuator (Physik Instruments, Waldbronn, Germany) driven by a 400-mA ENV400 Amplifier (Piezosystem Jena, Jena, Germany). The voltage command was filtered with an 8-pole Bessel filter (Khron-Hite, Brockton, MA) at 50 kHz to eliminate residual pipette resonance. Hair bundle deflections were monitored using a C2400 CCD camera (Hamamatsu). The 10–90% rise time of the probe was ∼20 µs.
